# Implementation of a High-Throughput Screen for Identifying Small Molecules to Activate the Keap1-Nrf2-ARE Pathway

**DOI:** 10.1371/journal.pone.0044686

**Published:** 2012-10-08

**Authors:** Kai Connie Wu, Peter R. McDonald, Jie Jerry Liu, Rathnam Chaguturu, Curtis D. Klaassen

**Affiliations:** 1 Department of Pharmacology, Toxicology, and Therapeutics, University of Kansas Medical Center, Kansas City, Kansas, United States of America; 2 University of Kansas High Throughput Screening Laboratory, University of Kansas, Lawrence, Kansas, United States of America; 3 Department of Internal Medicine, University of Kansas Medical Center, Kansas City, Kansas, United States of America; 4 Center for Advanced Drug Research, SRI International, Harrisonburg, Virginia, United States of America; University of Colorado Denver, United States of America

## Abstract

Nuclear factor erythroid 2-related factor 2 (Nrf2) is a transcription factor that induces a battery of cytoprotective genes involved in antioxidant defense through binding to Antioxidant Response Elements (ARE) located in the promoter regions of these genes. To identify Nrf2 activators for the treatment of oxidative/electrophilic stress-induced diseases, the present study developed a high-throughput assay to evaluate Nrf2 activation using AREc32 cells that contain a luciferase gene under the control of ARE promoters. Of the 47,000 compounds screened, 238 (top 0.5% hits) of the chemicals increased the luminescent signal more than 14.4-fold and were re-tested at eleven concentrations in a range of 0.01–30 µM. Of these 238 compounds, 231 (96%) increased the luminescence signal in a concentration-dependent manner. Chemical structure relationship analysis of these 231 compounds indicated enrichment of four chemical scaffolds (diaryl amides and diaryl ureas, oxazoles and thiazoles, pyranones and thiapyranones, and pyridinones and pyridazinones). In addition, 30 of these 231 compounds were highly effective and/or potent in activating Nrf2, with a greater than 80-fold increase in luminescence, or an EC50 lower than 1.6 µM. These top 30 compounds were also screened in Hepa1c1c7 cells for an increase in Nqo1 mRNA, the prototypical Nrf2-target gene. Of these 30 compounds, 17 increased Nqo1 mRNA in a concentration-dependent manner. In conclusion, the present study documents the development, implementation, and validation of a high-throughput screen to identify activators of the Keap1-Nrf2-ARE pathway. Results from this screening identified Nrf2 activators, and provide novel insights into chemical scaffolds that might prevent oxidative/electrophilic stress-induced toxicity and carcinogenesis.

## Introduction

Oxidative stress is the consequence of imbalanced production of reactive oxygen species (ROS) and the ability of cells to detoxify these ROS, which can result in ROS-induced tissue damage. In humans, oxidative stress is involved in the pathogenesis of numerous clinical conditions, including atherosclerosis [1], Alzheimer's disease [2], and rheumatoid arthritis [3]. In addition, ROS, together with other electrophiles, are capable of attacking DNA in the nucleus increasing the risk of carcinogenesis [4].

The Kelch-like ECH-associated protein 1 (Keap1) - nuclear factor, erythroid derived 2, like 2 (Nrf2) pathway serves as one of the major protective mechanisms in cells in response to oxidative/electrophilic stress. Under basal conditions, Nrf2 is sequestered in the cytoplasm by the cytoskeletal anchoring protein Keap1, and is targeted for ubiquitin-mediated proteasome degradation. Upon the stimuli of oxidative/electrophilic stress, Nrf2 is released from Keap1 and Nrf2 translocates into the nucleus [5]. Once in the nucleus, Nrf2 heterodimerizes with a variety of transcriptional regulatory proteins, including members of the activator protein-1 family (Jun and Fos), and the small Maf family of transcription factors [6]. These protein complexes bind to antioxidant response elements (ARE) located in the upstream promoter region of a battery of genes, and drives their transcription [7].

The Nrf2 target genes are involved in a variety of cytoprotective events, such as glutathione (GSH) synthesis and recycling (Gclc, Gclm, Gss, Gsr), reduction of hydrogen peroxide (Gpx), reduction of oxidized protein (Txn, Txnrd, Srxn), detoxification of electrophiles (Nqo1, Gst), and excretion of GSH-conjugated electrophiles (Mrp) [8]. Thus, it is not surprising that Nrf2 deficient mice are more susceptible, whereas Nrf2 enhanced mice are resistant to chemical-induced oxidative/electrophilic stress and subsequent tissue injury. For example, compared with wild-type mice, Nrf2-null mice are more susceptible to acetaminophen-induced liver injury [9], cigarette smoke-induced lung injury [10], dextran sulfate sodium/azoxymethane-induced colitis and colorectal cancer [11], and benzo[a]pyrene-induced forestomach cancer [12]. In contrast, Keap1-knockdown and Keap1-hepatoctye knockout mice, in which Nrf2 is constitutively activated, are highly resistant to acetaminophen [13], diquat [14], and cadmium [15]-induced lethality and tissue injury. In addition, a number of synthetic and natural compounds protect against oxidative/electrophilic stress-induced toxicity, at least partially through activating Nrf2. For example, curcumin protects against focal ischemia of the cerebrum through upregulation of Nrf2 [16], and oltipraz protects against ANIT-induced cholestasis through Nrf2 activation [17].

These data suggest potential therapeutic applications of the Keap1-Nrf2 pathway, and thus Nrf2 is a promising drug target in the treatment of oxidative/electrophilic stress-induced diseases. A quantitative bioassay evaluating the induction of NAD(P)H:quinone oxidoreductase 1 (Nqo1), the prototypical Nrf2 target gene, in Hepa1c1c7 murine hepatoma cells was developed and still remains a major screening tool for potential activators of the Keap1-Nrf2 pathway [18]. To date, a number of compounds with diverse chemical structures have been shown to activate Keap1-Nrf2, including oxidizable diphenols (tBHQ), dithiolethiones (oltipraz), isothiocyanates (sulforaphane), and Michael acceptors (curcumin, cinnamates, and chalcones) [19]. In an effort to develop more potent and effective activators of the Keap1-Nrf2 pathway, chemical derivatives of known active compounds were synthesized and screened. The most potent known Nrf2 activator, 2-cyano-3,12-dioxoolean-1,9-bien-28-oic acid imidazole (CDDO-Im), is a semisynthetic triterpenoids derived from oleanolic acid [20]. Based on the structure-activity relationship analyses of the oleanolic triterpenoids, (±)-(4bS,8aR,10aS)-10a-ethynyl-4b,8,8-trimethyl-3,7-dioxo-3,4b,7,8,8a,9,10,10a-octahydrophenanthrene-2,6-dicarbonitrile (TBE-31), was synthesized. Both CDDO-Im and TBE-31 activate the Keap1-Nrf2 pathway at nano-molar concentrations *in vitro and in vivo* [21].

Despite the discovery of a few potent Nrf2 activators (CDDO compounds and TBE-31), there is limited information about the chemical scaffolds that can potentially activate Nrf2. Recently, AREc32 cells were engineered. AREc32 cells, which are derived from MCF7 human breast cancer cells, are stably transfected with a luciferase reporter gene construct under the control of eight copies of rat Gsta2 AREs in the promoter region [22]. The AREc32 cells provide a rapid and convenient quantification of Nrf2-ARE induction by chemicals, and makes large scale-screening of Nrf2 activators possible.

The aim of the present study was to develop a high-throughput assay to evaluate Nrf2 activation using the AREc32 cells, screen a library of 47,000 compounds, and to find compounds that are potent and effective activators of Nrf2. In addition, through structural activity relationship analyses, the present study also aimed to discover novel chemical scaffolds that are likely to activate the Keap1-Nrf2-ARE pathway. Results from this screening identified strong Nrf2 activators, and provide novel insights into chemical scaffolds that might detoxify oxidative/electrophilic stress and prevent oxidative/electrophilic stress-induced toxicity and carcinogenesis.

## Materials and Methods

### Cell growth and maintenance

The AREc32 cells were obtained from CRX biosciences (Dundee, Scotland, UK). The AREc32 are a stable cell line derived from the human MCF7 breast carcinoma cell line with a transfected luciferase gene construct that under the control of eight copies of rat Gsta2 AREs in the promoter region [23]. AREc32 cells were maintained in Dulbecco's Modified Eagle's medium (DMEM) containing glutamax supplemented with 10% fetal calf serum and the antibiotic G418 (Life Technologies Corporation, Carlsbad, CA). The cells were grown at 37°C in the presence of 5% CO_2_.

AREc32 cells were seeded into 384-well plates (flat-bottom white, opaque, sterile, with lids) at a density of 3,500 cells/well using a Wellmate bulk dispenser (Thermo Fisher Scientific, Waltham, MA) in 50 µL of complete media per well. Cell plates were incubated at room temperature for 30 min following seeding to allow for even cell settling. Cell plates were then incubated at 37°C, 5% CO_2_ in a 95% humidified incubator for 20 hrs.

Murine hepatoma Hepa1c1c7 cells were obtained from ATCC (Manassas, VA) and maintained in DMEM with glutamate, supplemented with 10% (v/v) heat-inactivated FBS, penicillin (100 units/ml), and streptomycin (100 µg/ml). The cells were maintained at 37°C in the presence of 5% CO_2_.

### Compound libraries and preparation

Four libraries of compounds were screened for Nrf2 activation in the present study: 2000 compounds were obtained from MicroSource Discovery Systems (www.msdiscovery.com/spectrum.html), 1120 compounds were obtained from Prestwick Chemical Library (Prestwick Chemical, Washington, DC), 1920 compounds were obtained from the University of Kansas Center of Excellence in Chemical Methodologies & Library Development (KU-CMLD), and 41,888 compounds were obtained from ChemBridge Small Molecule Library (ChemBridge Corporation, San Diego, CA).

The four libraries of compounds were stored at 2859 µM in 100% DMSO, and 175 nL of each compound was transferred to the 50 µL cell culture medium in the receiving well. Chembridge library compounds were dispensed by the Matrix PlateMate Plus automated nanoliter capacity liquid handler (Thermo Fisher Scientific, Waltham, MA), followed by three gentle mixings. Compounds from MicroSource, Prestwick, and CMLD libraries, as well as the compounds for the concentration-response validation, were dispensed by Labcyte Echo 550 Compound Reformatter (Labcyte Inc., Sunnyvale, CA), which allows the accurate transfer of small volumes of liquid. The final concentration of each chemical in the full library screen was 10.0 µM, with a DMSO content of 0.35%.

### Quantification of ARE activation by Promega Steady-Glo luciferase assay system

AREc32 cells were exposed to library compounds for 24 hrs at 37°C, 5% CO_2_ in a 95% humidified incubator, then removed from the incubator and left at room temperature for 20 min to equilibrate the plate and its contents to room temperature. The Matrix Wellmate dispensed Steady-Glo luciferase assay reagent (Promega, Madison, WI) to all cells, 10 µL per well, and plates were shaken for 1 min at 1600 rpm. The luminescence intensities were read 30 min later on a Tecan Safire2 microplate reader (Männedorf, Switzerland). The luminescence values used for data analysis were derived from a luciferase reaction (Figure S1). The Steady-Glo reagent produces cell lysis and generation of a luminescent signal, which is proportional to ARE activation, via the luciferase reporter in the AREc32 cell line.

Four controls were used on each plate of cells: (1) cells treated with tBHQ, a known ARE activator (positive control), (2) cells treated with CDDO-Im, a very potent activator of Nrf2/ARE (positive control), (3) cells in media containing 0.35% DMSO (vehicle control), and (4) cells in media containing no DMSO (control cells) to measure background luminescence. The plate map for the controls is displayed in Figure S2.

### Z′ factor for Pass/Fail Criterion

The positive and negative controls were required to assure uniformity from plate to plate, and from screening batch to batch. The controls were used to calculate a Z′ factor value for each plate, a measure of assay robustness and variability popularly used for high throughput screening. The Z′ factor compares the baseline background (minimum ARE signal) from the DMSO vehicle control, and the maximum signal of response of the positive controls tBHQ and CDDO-Im [24], [25]. The Z′ factor formula relies on the mean and standard deviation of the maximum signal and the minimum baseline, as shown in Figure S3. In this assay, screening plates were expected to have a Z′ value equal to or greater than 0.6. Plates with Z′ values below 0.4 were individually investigated, and rejected or repeated on a plate by plate basis.

### Quantification of Nqo1 mRNA in Hepa1c1c7 cells

The top 30 hits from the primary screen were further validated by quantifying the mRNA of Nqo1, a prototypical Nrf2 target gene, in Hepa1c1c7 cells by real time-PCR. Cells were grown in 24-well plates at a density of 30,000 cells per well for 12 hrs, and subsequently incubated with test compounds at 6 concentrations (0.1–3 µM) for 24 hrs. After incubation, the medium was decanted and total RNA was isolated using RNAzol B reagent (Tel Test, Inc., Friendswood, TX). cDNA was synthesized with a High Capacity cDNA Archive Kit (Applied Biosystems, Foster City, CA) from total RNA, and the resulting cDNA was used for real-time PCR to quantify Nqo1 mRNA, with β-actin used as the internal control. The primer sequences for Nqo1 and β-actin are listed in Table S1.

### Compound structure clustering analysis

Preliminary hit clustering was based on EC50 values from the concentration-response curves of the top 240 compounds, and accomplished via the Selector program from Tripos via the Jarvis Patrick routine, using default parameters. From each preliminary cluster, the largest conserved substructure present in at least half of the cluster members was identified. Each cluster was then manually edited to remove compounds that did not contain the largest conserved substructure identified in the previous step. Compounds that had not originally been selected to a given cluster but containing the cluster's characteristic conserved substructure were then added to the cluster.

### Induction of Nqo1 by the top 30 hits from the primary screening in Hepa1c1c7 cells

Hepac1c7 cells were seeded into 24-well plates at a density of 30,000 cells/well in 1 mL complete media per well and cultured overnight to allow attachment. Cells were treated the next day with the top 30 compounds from the primary screening and were harvested 24 h after treatment. Total RNA samples were isolated by using RNAzol B reagent (Tel-Test, Inc., Friendswood, TX) according to the manufacturer's protocol, and were reverse-transcribed into cDNA by High Capacity cDNA Archive Kits (Applied Biosystems, Foster City, CA). The resulting cDNA was used for real-time PCR analysis using SYBR® Green PCR Master Mix in a 7900HT Fast Real-Time PCR System (Applied Biosystems, Foster City, CA). Oligonucleotide primers specific to mouse β-actin and Nqo1 are shown in Table S1.

## Results

### Luciferase assay signal stability

**Figure 1:**
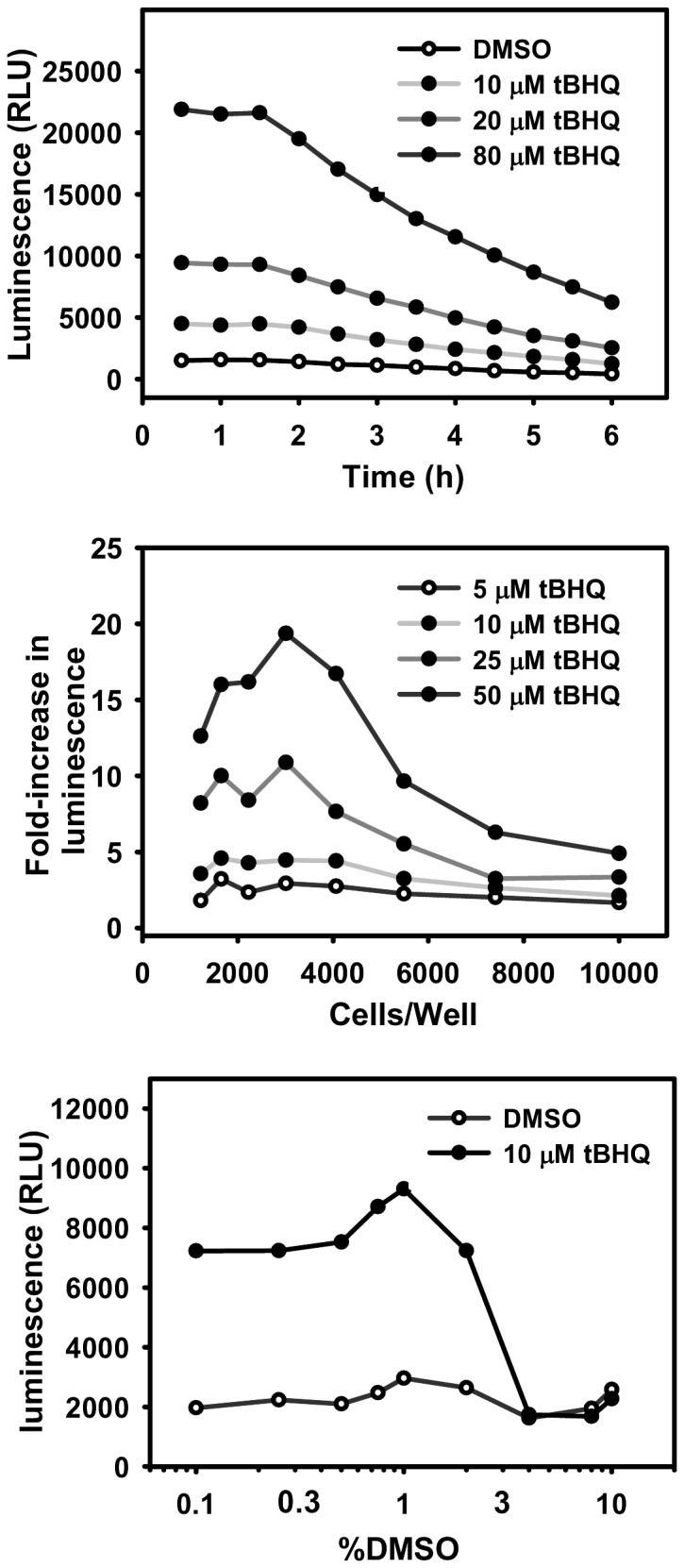
Development and optimization of an ARE induction assay in AREc32 Cells. (A) The luciferase assay signal stability in the presence of 0, 10, 20, or 80 µM tBHQ.(B) The effect of AREc32 cells seeding density on the luciferase assay sensitivity in the presence of 0, 10, 20, or 80 µM. (C) The effect of DMSO concentration on the luciferase assay sensitivity using a range of 0%–10% DMSO in the presence or absence of 0 or 10 µM tBHQ.

Batch processing of 384-well plates for the HTS library screen requires the readout signal to be steady for at least 30 min, and preferably one hr to minimize timing effects. To test the luciferase assay signal stability of AREc32 cells, they were seeded at 3,000 cells/well in a 384-well plate, and tBHQ, a typical Nrf2 activator, was added to cells to make final concentrations of 0, 10, 20, or 80 µM. All wells had a final DMSO concentration of 0.5%. Twenty-four hrs after treatment, luciferase activity was assessed using the Steady-Glo luciferase assay with luminescence readout. The luminescence signal was recorded 30 min after cell lysis, and was quantified repeatedly every 30 min for six hrs. As shown in Fig. 1A, the luminescent signal was strong and stable for over one and half hrs after adding the luciferase reagent.

### Optimization of AREc32 cells seeding density

To determine the optimal seeding density of AREc32 cells under screening conditions, cells were seeded at a range of 1,000–10,000 cells/well in a 384-well plate, and were treated with tBHQ or DMSO vehicle (0–50 µM tBHQ, 0.5% DMSO). Twenty-four hrs after treatment, luciferase activity was assessed with luminescence readout. The luminescent signal was quantified 30 min after adding the luciferase reagent to the cells. Data is presented as fold increase in luminescence by tBHQ over vehicle control. As shown in Fig. 1B, 3,000–3,500 cells/well provided optimal activation of the ARE-luciferase construct by tBHQ.

### The effect of DMSO on cell viability and assay sensitivity

Because the compounds are maintained in 100% DMSO, the effect of DMSO on viability and assay sensitivity of AREc32 cells was investigated. Cell viability was unaffected by DMSO below 0.75% (data not shown). To test the effect of DMSO on assay sensitivity, cells were seeded at 3,000 cells/well in a 384-well plate and a mixture of media and DMSO was added to each well to achieve a final DMSO content of 0%–10%. Additionally, 5 µL of media with or without tBHQ was added immediately following addition of DMSO. Twenty-four hrs after treatment, luciferase activity was assessed by the luminescence readout. As shown in Fig. 1C, the ability of tBHQ to activate the luciferase reporter construct was hindered by DMSO concentration above 1%. To avoid cell stress that may activate undesired molecular pathways, 0.5% DMSO was selected as the maximum tolerable concentration of DMSO in the cell culture medium.

### Dose-response activation of ARE-luciferase reporter construct in AREc32 cells by known Nrf2 activators

**Figure 2:**
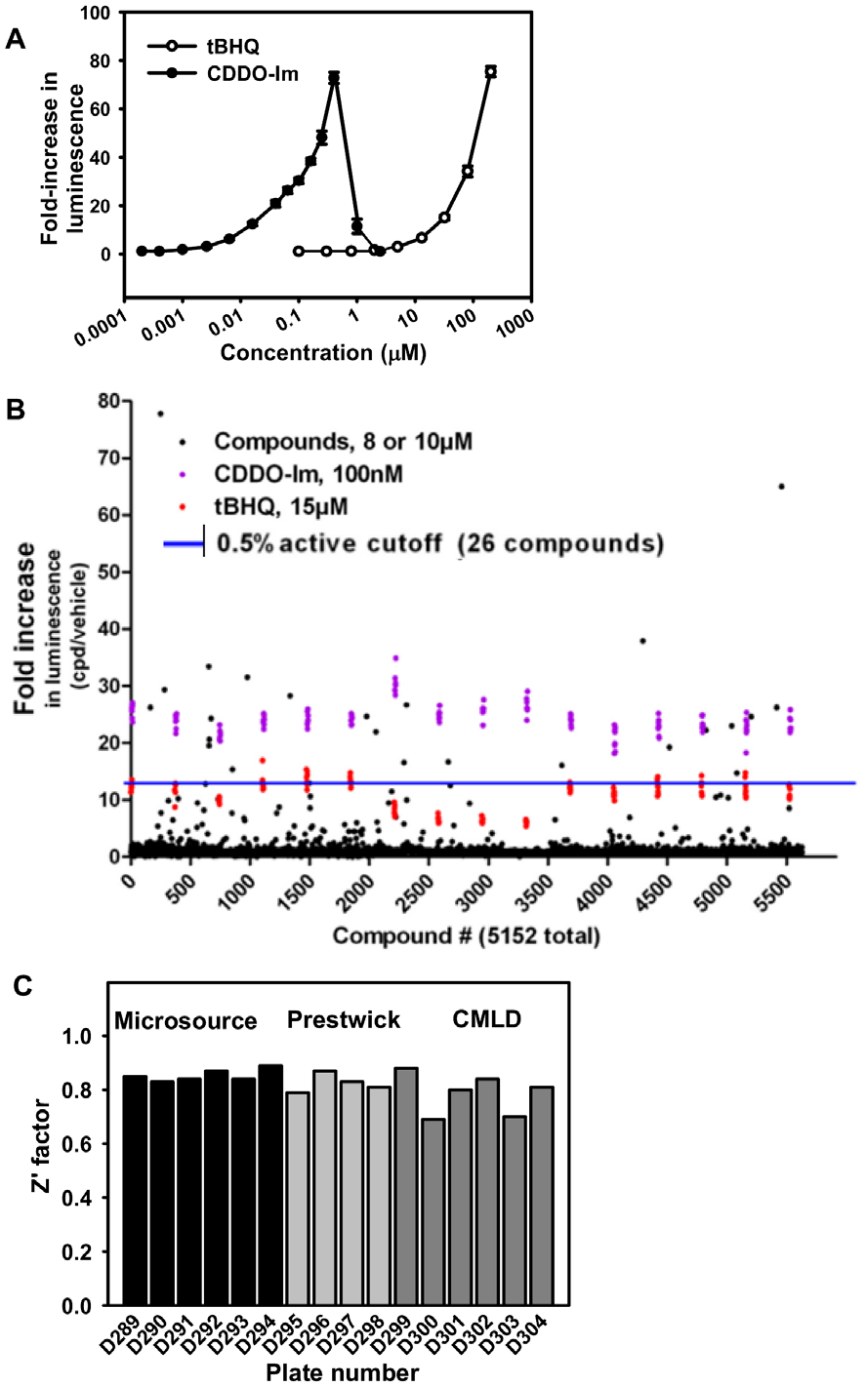
Validation of the ARE induction assay using known Nrf2 activators and through pilot screening. (A) Concentration-response curves of tBHQ and CDDO-Im to increase the luminescent signal in AREc32 cells. (B) Activity spread of compounds in Microsource, Prestwick, and CMLD libraries together with positive controls. Compounds in Microsource and CMLD libraries were tested at 10 µM, and compounds in Prestwick library were tested at 8 µM. (C) Z′ scores for 16 pilot screening plates.

To validate the ARE-luciferase reporter assay, tBHQ and CDDO-Im, two prototypical Nrf2 activators were tested in the AREc32 cells. Cells were seeded at 3,000 cells/well in a 384-well plate and treated with DMSO vehicle or compound (0–100 µM for tBHQ and 0–2 µM for CDDO-Im). Twenty-four hrs after treatment, luciferase activity was assessed by quantifying the luminescent intensity. As shown in Fig. 2A, both tBHQ and CDDO-Im increased the luminescence signal in a dose-dependent manner, with over a 70-fold increase in luminescence at 100 µM (tBHQ) or 300 nM (CDDO-Im).

Other known Nrf2 activators, namely curcumin, sulforaphene, and genistein, also increased the luminescence signal in AREc32 cells in a concentration-dependent manner (data not shown).

### Pilot screening of 5040 compounds from the Microsource, Prestwick, and CMLD libraries

To further validate the ARE-luciferase reporter assay in a high-throughput manner, 1,120 compounds from the Prestwick library were screened in the AREc32 cells at seven concentrations and within a range of 0.25–16 µM. Twenty-one compounds (top 1.9%) increased the luminescence signal in a concentration-dependent manner, with the maximum fold-induction value higher or comparable to tBHQ (data not shown). The 2,000 compounds from the Microsource library and 1920 compounds from the CMLD library were screened at a final concentration of 10 µM. The fold-induction of the luminescent signals of the tested compounds is shown in Fig. 2B, with the fold-induction values of positive controls for comparison. Specifically, the fold-induction values of compounds in Microsource and CMLD libraries were tested in a final concentration of 10 µM, and compounds in the Prestwick library were treated at 8 µM.

The Z′ factor measure of assay robustness and variability was plotted for the 16 validation library plates (Fig. 2 C). The Z′ scores for the validation library screening plates of libraries confirmed the Z′ score of the screen was greater than 0.6 for all plates, confirming the quality of the assay methodology.

### Full screening of 47,000 compounds from the Microsource, Prestwick, and Chembridge Libraries

**Figure 3:**
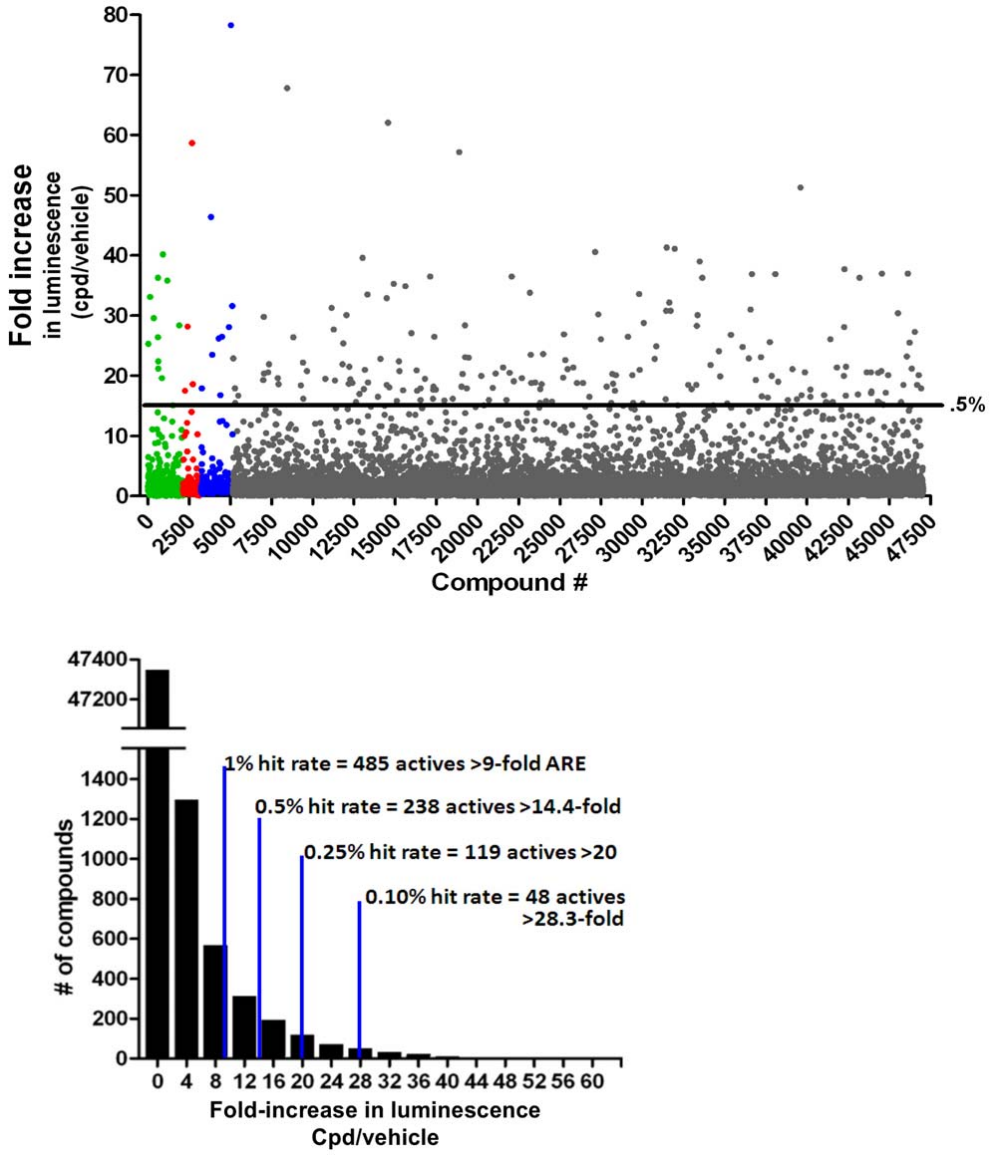
Full library screening for Nrf2 activators through ARE induction assay in AREc32 cells. (A) The scatterplot distribution of the screening actives from the ARE library screen was calculated using data from the Chembridge, Prestwick, Microsource, and CMLD library compounds. The fold increase in luminescence, indicative of ARE activation by compounds in AREc32 cells, is plotted against each individual well of the libraries. (B) Frequency distribution of the Chembridge, Prestwick, Microsource, and CMLD library compounds to increase the luminescent signal in AREc32 cells.

After the luciferase-based reporter assay was validated in a high-throughput system, the full library containing 47,000 compounds were screened using this assay. The fold increase in luminescence, indicative of Nrf2 activation by compounds in AREc32 cells, was plotted against each individual well of the libraries (Fig. 3A). The majority of compounds did not activate Nrf2, and were densely packed at the bottom of the scatterplot. Only the top 0.5% hits (238 compounds) increased the luminescence more than 14.4-fold. A histogram summarizing the frequency distribution of the ability of the compounds to increase luminescence over the DMSO control is shown in Fig. 3B. The majority of compounds did not activate Nrf2. The top 1% hits (485 compounds) increased the luminescence more than 9-fold, the top 0.5% hits (255 compounds) increased the luminescence more than 14.4-fold, the top 0.25% hits (119 compounds) increased the luminescence more than 20-fold, and the top 0.1% hits (48 compounds) increased the luminescent signal more than 28-fold.

### Dose-response curves for the 4 compounds with the highest maximal ARE activation

**Figure 4:**
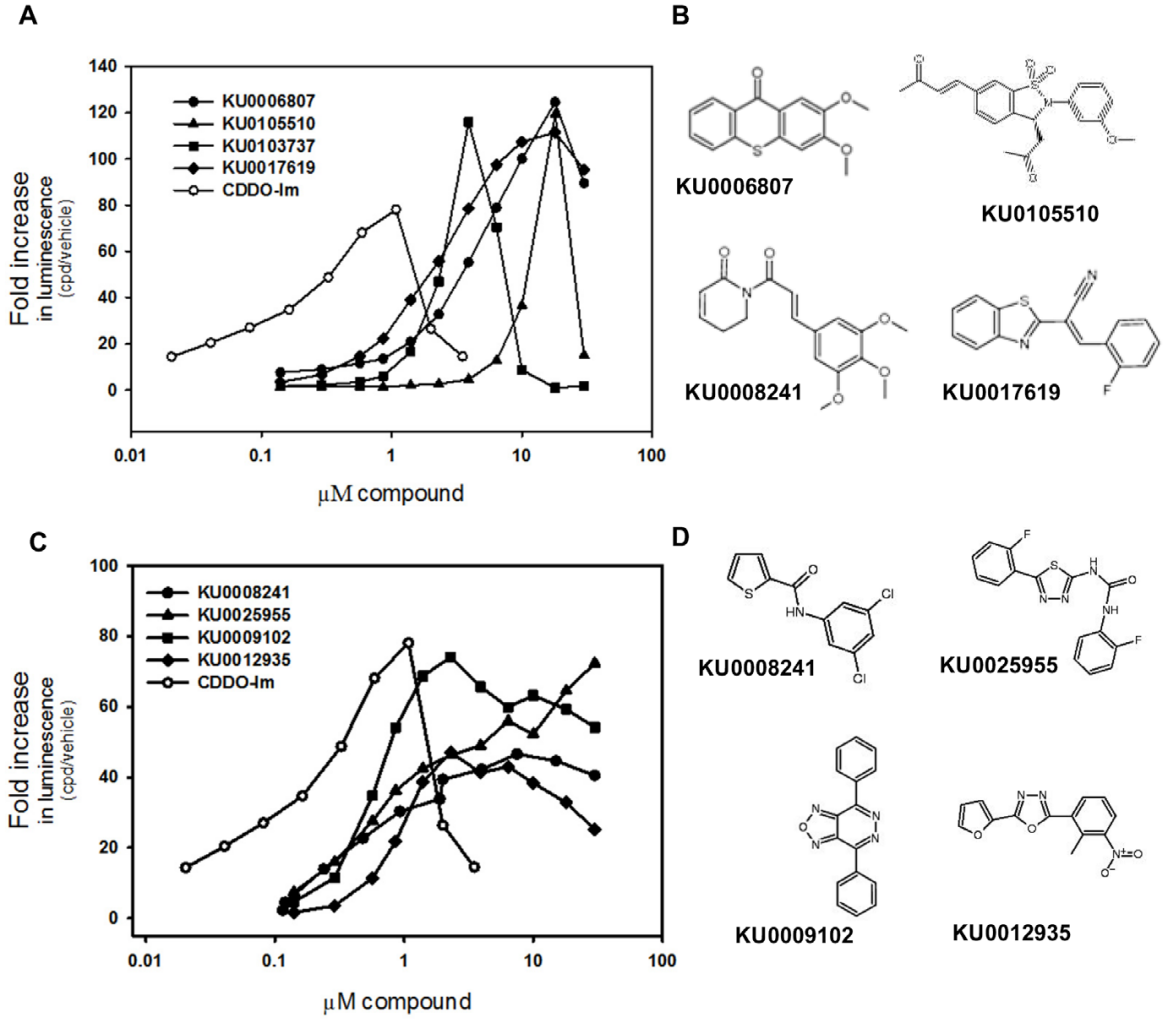
Concentration-response curves of the most effective and potent compounds. (A) Concentration-response curves and (B) chemical structures of top four compounds with greatest maximum fold-increase in luminescent signal. (C) Concentration-response curves and (D) chemical structures of top four compounds with lowest EC50. AREc32 cells were treated with compound (0.01–30 µM) or DMSO vehicle. 24 hours after treatment, luciferase activity was assessed using the Steady-Glo luciferase assay with luminescence readout. The luminescence of each well was divided by the median luminescence of the DMSO vehicle control wells to generate the fold ARE activation.

The 255 most active compounds (top 0.5% hit) from the full library screening were retested at multiple concentrations (0.14–30 µM), and 91% of them (247 compounds) activated the Nrf2 pathway in a concentration-dependent manner. Among those 247 validated hits, 18 compounds were shown to be extremely effective and each produced a maximum fold-activation higher than that of CDDO-Im. The concentration-response curves of the top 4 compounds are shown in Fig. 4A. Compound KU0006807 increased the luminescence signal 125-fold at 18 µM; KU 0105510 increased the luminescence signal 119-fold at 18 µM; KU0103737 increased the luminescence signal 115-fold at 3.9 µM; and KU0017619 increased the luminescence signal 111-fold at 18 µM. The chemical structures of KU0006807, KU 0105510, KU0103737, and KU0017619 are shown in Fig. 4B.

### Dose-response curves for the 4 compounds with the lowest EC50 for Nrf2-ARE activation

Among those 247 validated hits from screening the full library, 6 compounds were shown to be extremely potent and had EC50 values lower than 1 µM. However, none of the compounds tested had an EC50 value lower than that of CDDO-Im. The concentration-response curves of the top 4 compounds are shown in Fig. 4C. KU0009102 had an EC50 value as 0.7 µM, and maximum Nrf2 activation of 74-fold; KU0008241 had an EC50 value as 0.9 µM, and maximum Nrf2 activation of 46-fold; KU0025955 had an EC50 value as 0.9 µM, and maximum Nrf2 activation of 72-fold; and KU0012935 had an EC50 value as 1 µM, with maximum Nrf2 activation of 47-fold. The chemical structure of KU0009102, KU0008241, KU0025955, and KU0012935 are shown in Fig. 4D.

### Structure clusters of hits from the primary screening

**Figure 5:**
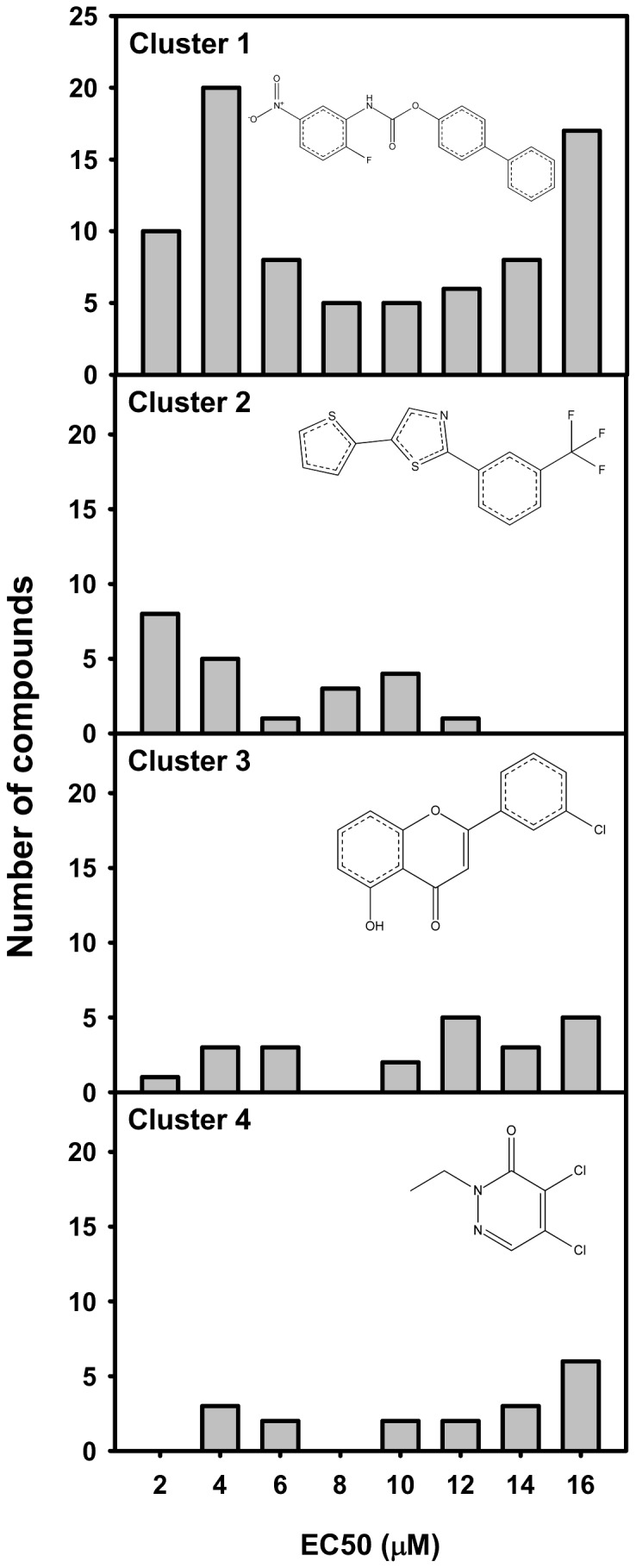
Chemical scaffolds clustered in the top 247 validated hits from the primary screening. Preliminary hit clustering was based on the EC50 value from the concentration-response curves of the top 240 compounds, and accomplished via the Selector program from Tripos via the Jarvis Patrick routine, using default parameters.

The chemical structures of the top 247 hits fall into four clusters. The chemical scaffolds of the clusters, as well as the compound numbers are noted in Fig. 5. Cluster 1 contains 80 structures that are related diaryl amides and diaryl ureas, and 10 of them were very potent with EC50 values lower than 2 µM. Cluster 2 contains 22 structure-related oxazoles and thiazoles, and eight of them were highly potent with EC50 values lower than 2 µM. Cluster 3 contains 23 structure-related pyranones and thiapyranones, including one highly potent compound with an EC50 lower than 2 µM. Cluster 4 contains 22 structure-related pyridinones, pyridazinones, and pyrimidones, but none had EC50 values lower than 2 µM.

### Induction of Nqo1 by the top 30 hits from the primary screening in Hepa1c1c7 cells

**Figure 6:**
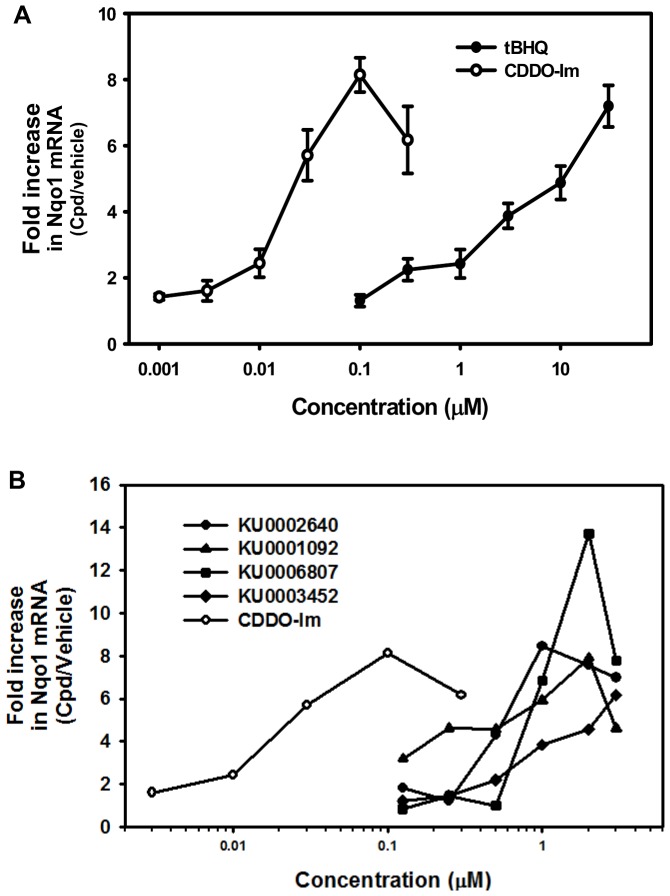
Concentration-response curves of known Nrf2 activators and most active compounds from the secondary screening. Concentration-response curves of (A) tBHQ and CDDO-Im, and (B) top 4 compounds from the secondary screening. Hepa1c1c7 cells were treated with compound (0.01–3 µM) or DMSO vehicle. 24 hours after treatment, mRNA of Nqo1 was quantified using reverse transcription q-PCR analysis. Fold-increase in Nqo1 mRNA was normalized by cells treated with DMSO vehicle.

To validate the active compounds using a different technique than the ARE-luciferase assay, and to determine the ability of active compounds to induce cytoprotective genes, a secondary screen assay was developed to quantify Nqo1 mRNA in Hepa1c1c7 cells. As shown in Fig. 6A, both tBHQ and CDDO-Im increased Nqo1 mRNA in a concentration-dependent manner, with over a 7-fold increase in Nqo1 mRNA with 30 µM tBHQ or 100 nM CDDO-Im.

Nineteen compounds with the lowest EC50 values and 14 compounds with the highest maximum increase of luminescence signal from the primary screen were selected for the secondary screen. Three compounds (KU0002640, KU0003452, and KU0013654) were shown to activate Nrf2 at both the lowest EC50 concentrations and the highest maximum increase of luminescence. Thus, 30 compounds were screened in Hepa1c1c7 cells at six concentrations (0–3 µM). Among those 30 compounds, 17 of them increased Nqo1 mRNA in a concentration-dependent manner, and the concentration-response curves of the most effective four compounds are shown in Fig. 6B. Specifically, KU002640, which has both the lowest EC50 and highest Nrf2 activation in the primary screen, also increased Nqo1 mRNA the most in the secondary screen.

## Discussion

The present study describes the development, implementation, and validation of a high-throughput screen to identify activators of the Keap1-Nrf2-ARE pathway. Using a luciferase-based assay driven by AREs in the promoter region of the luciferase gene, the AREc32 cells provides a rapid and convenient quantification of Nrf2-ARE induction by small molecules, and made large scale-screening for Nrf2 activators possible.

In contrast to many focused-screenings for Nrf2 activators in previous reports, which compared the efficacy and potency of potential Nrf2 activators that are derived from a single chemical scaffold [26], [27], [28], the present study screened 47,000 chemicals with diverse sources and chemical structures. This experimental design increased the chances of identifying novel chemical scaffolds for analogs that could potentially be further developed to have improved potency, efficacy, and pharmacokinetics. The present study summarizes four novel chemical scaffolds that clustered in the top 0.5% hits: (1) diaryl amides and diaryl ureas, (2) oxazoles and thiazoles, (3) pyranones and thiapyranones, and (4) pyridinones and pyridazinones. Among these four chemical clusters, cluster 1 contains the greatest number of the top 0.5% hits (80 hits in cluster 1, 22 hits in cluster 2, 23 hits in cluster 3, and 22 hits in cluster 4), and contains the most number of hits with EC50 values less than 1.4 µM (5 hits in cluster 1, 4 hits in cluster 2, and no hits in cluster 3 or cluster 4). Thus, the chemical scaffold of cluster 1 may have the greatest potential for designing chemical analogs and to develop strong Nrf2 activators.

The present study validates the top 0.5% hits (238 compounds) with eleven concentrations in a wide range of concentrations. Thus, the EC50 value for activating Nrf2 of each compound is available, which makes the comparison of the potency of the compounds possible. In addition, the concentration-response assay also generates the highest maximum fold-increase in luminescence, and the ranking of the top hits was modified accordingly. For example, KU0105510 was shown to increase the luminescence 26-fold at 10 µM. However, the concentration-response assay shows that KU0105510 can increase the luminescence 119-fold at 18 µM, which makes it the second most effective hit in the full library (Fig. 4A). Some extremely potent compounds activated Nrf2 at low concentrations, and the activity decreased at higher concentrations. For example, KU0103737 increased the luminescent signal by 18-fold at 10 µM. However, the concentration-response assay shows that KU0103737 induced the luminescent signal most at 3.9 µM (116-fold), and the induction was blunted at higher concentrations (Fig. 4). Collectively, the concentration-response assay identified extremely effective and potent compounds among the top 0.5% hits.

The concentration-response studies also revealed two distinct patterns in Nrf2-ARE induction. The first battery of hits (example: KU0103737 and KU0105510) increased the luminescent signal markedly (over 110-fold), but the increase was blunted at higher concentrations after reaching the maximum fold-increase. The second battery of hits (example: KU0009102 and KU0003004) increased the luminescence moderately (70-fold), reached a plateau, but also increased the luminescence moderately (60–70 fold) at higher concentrations (6–30 µM). Compared with the first battery of hits, the second battery of hits may be more plausible for drug development to have a steady effect over a wide range of drug concentrations.

For luciferase reporter-based assays, one major concern is identifying false active compounds that increase the luminescent signal not through activation of the target, but through stabilization of the luciferase enzyme. Therefore, a counter screen was performed to rule out such false positive hits. The 196 hits from the primary screen were randomly selected and tested for the capability of stabilizing purified firefly luciferase. Among these 196 hits, only 11 hits tested stabilized luciferase enzyme, and thus could be false positives (data not shown).

To investigate whether the validated hits from the primary screen can also induce Nrf2 target genes, a secondary screen was designed to test the ability of the hit compounds to induce Nqo1, the prototypical Nrf2 target gene, in Hepa1c1c7 cells. The results shows that 17 of the 30 compounds increase Nqo1 mRNA in a concentration-dependent manner. The relatively low validation rate may result from four reasons. First, once Nrf2 translocates into the nucleus upon activation, Nrf2 heterodimerizes with other transcription factors (example: small Mafs, c-Jun, and c-Fos) and Nrf2-ARE signaling is affected by these Nrf2-binding transcription factors. Recently, ERα was shown to bind Nrf2 in the nucleus and suppress Nrf2-dependent gene transcription [29]. Thus, the different responses of AREc32 cells and Hepa1c1c7 cells to Nrf2-ARE activation may result from distinct estrogen signaling in these two cell lines (human breast cancer cells versus mouse hepatoma cells). Secondly, the luciferase gene in AREc32 cells contains eight AREs in the promoter region [23]. However, mouse Nqo1 gene is induced through one functional ARE [30]. Thus, the secondary assay may be less sensitive to Nrf2 activators than the primary assay. Lastly, the AREc32 and Hepa1c1c7 cells may have distinct expression of uptake transporters, resulting in different bioavailability of the test compounds.

**Figure 7:**
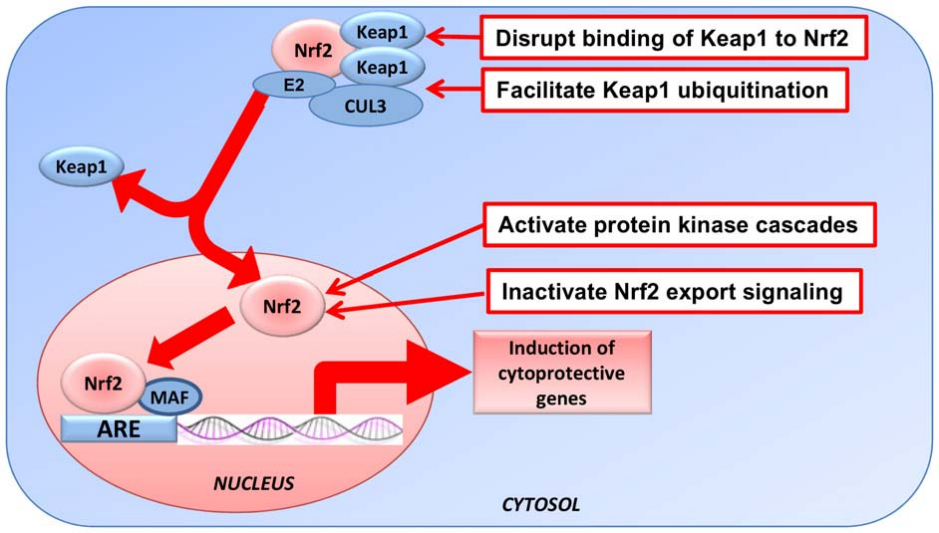
Hypothetical modes of action of actives compounds. Four potential mechanisms of action for hit compounds to activate Keap1-Nrf2-ARE pathway. Hit compounds can disrupt binding of Keap1 to Nrf2, facilitate Keap1 ubiquitination, inactivate Nrf2 export signaling, or activate protein kinase cascades for Nrf2 phosphorylation.

As for all transcription activation-based assays, neither the primary nor the secondary screening assay provide information about how active compounds activate the Keap1-Nrf2-ARE pathway. However, previous reports suggest that these active compounds may activate the Keap1-Nrf2-ARE pathway through multiple mechanisms [31], as shown in Fig. 7. Some active compounds may disrupt binding of Keap1 to Nrf2, leading to the release of Nrf2, and allowing Nrf2 to translocate to the nucleus [32]. Some active compounds may lead to ubiquitination of Keap1 instead of Nrf2, and facilitate Nrf2 accumulation [33]. Some active compounds may cause an inactivation of the Nrf2 export signal, increasing Nrf2 accumulation in the nucleus [34]. Some active compounds may increase nuclear export of Bach1, which competes with Nrf2 for small Maf binding [35]. Lastly, some active compounds may activate protein kinase cascades (example: MAPK and PI3K) [36], causing enhanced Nrf2 phosphorylation.

In conclusion, the present study documents the development, implementation, and validation of a high-throughput screen to identify activators of the Keap1-Nrf2-ARE pathway. Eight compounds that are extremely potent and effective in activating Nrf2 were identified. In addition, the present study also summarized four novel chemical scaffolds that may have utility in rational design of Nrf2-activating compounds for therapy of oxidative/electrophilic stress-induced diseases.

## Supporting Information

Figure S1 **Experimental design for primary screening.** The AREc32 cell line was exposed to library compounds for 24 hours at, then removed from the incubator and left at room temperature for 20 minutes to equilibrate the plate and its contents to room temperature. The Matrix Wellmate dispensed Steady-Glo luciferase assay reagent to all cells, 10 µL per well, and plates were shaken for 1 minutes at speed 1600 rpm. 30 min later, the luminescence intensities were read on the Tecan Safire2 microplate reader. The luminescence values used for data analysis were derived a luciferase reaction.
(TIF)

Figure S2 **Plate design for primary screening.** No compounds were present in the first two columns of plates to allow room for in-plate controls. Grey: library compound containing wells. Glue: cells treated with 10 µM tBHQ. Pink: cells treated with 100 nM CDDO-Im. Orange: cells in media containing 0.35% DMSO. Yellow: cells in media containing no DMSO.
(TIF)

Figure S3 **Calculation formulation of Z′ factor.**
(JPG)

Table S1 **Oligonucleotide sequences for primers specific for mouse β-actin and Nqo1.**
(DOCX)
