# Supplementary figures and images for: Implementation of a High-Throughput Screen for Identifying Small Molecules to Activate the Keap1-Nrf2-ARE Pathway
Source: PLoS One. 2012 Oct 8;7(10):e44686. doi: 10.1371/journal.pone.0044686 (PMC3466241; doi:10.1371/journal.pone.0044686)

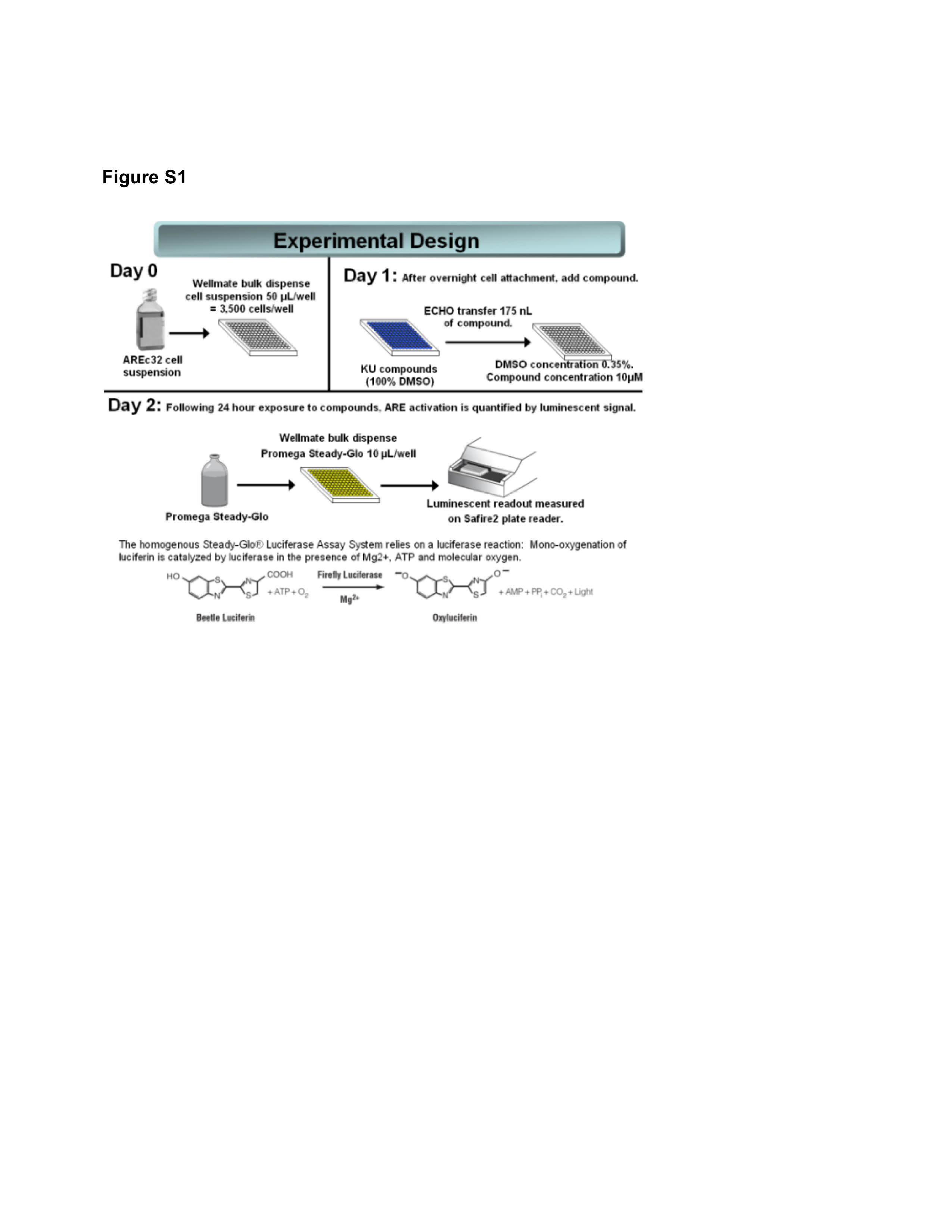

Supplement: Figure S1 — Experimental design for primary screening. The AREc32 cell line was exposed to library compounds for 24 hours at, then removed from the incubator and left at room temperature for 20 minutes to equilibrate the plate and its contents to room temperature. The Matrix Wellmate dispensed Steady-Glo luciferase assay reagent to all cells, 10 µL per well, and plates were shaken for 1 minutes at speed 1600 rpm. 30 min later, the luminescence intensities were read on the Tecan Safire2 microplate reader. The luminescence values used for data analysis were derived a luciferase reaction. (TIF) [file pone.0044686.s001.tif]

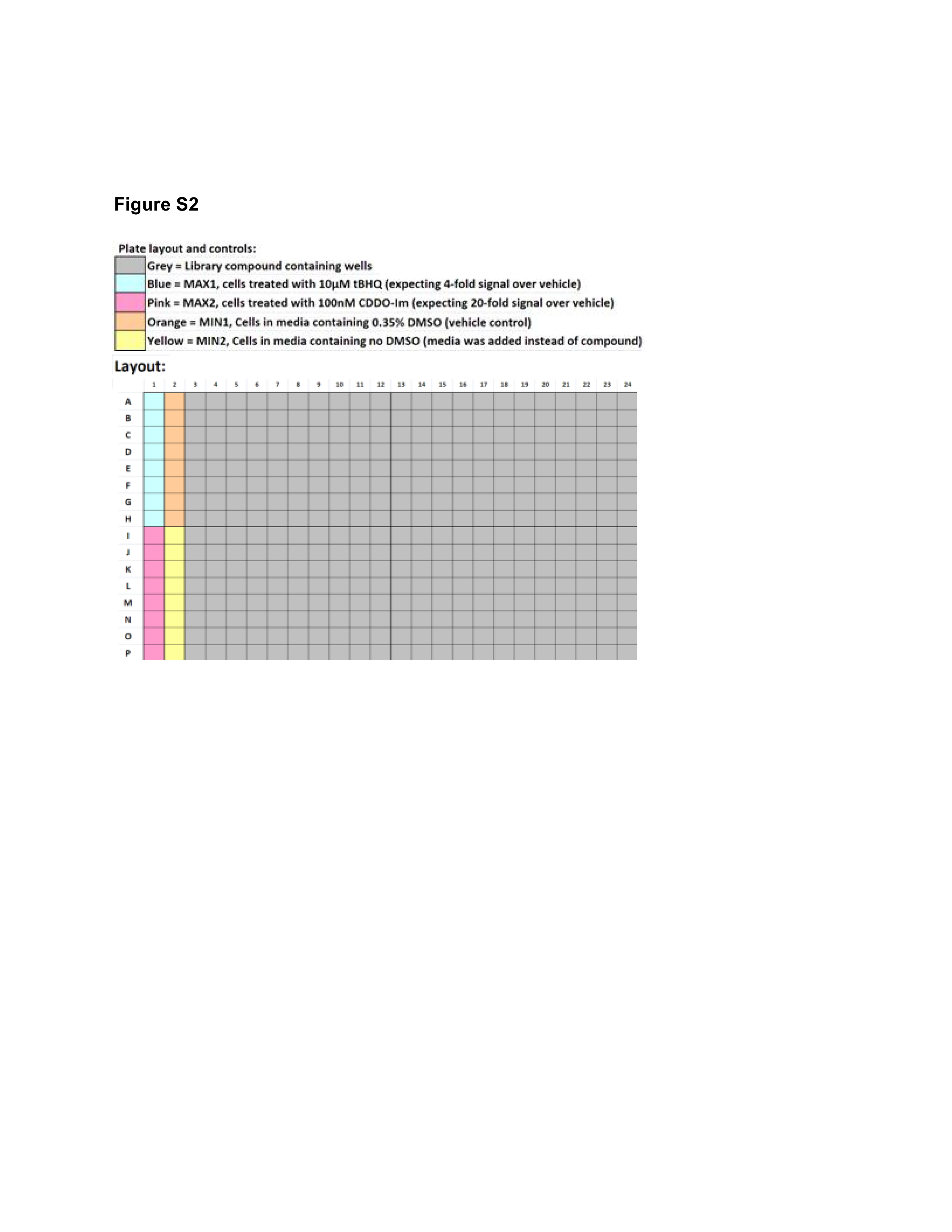

Supplement: Figure S2 — Plate design for primary screening. No compounds were present in the first two columns of plates to allow room for in-plate controls. Grey: library compound containing wells. Glue: cells treated with 10 µM tBHQ. Pink: cells treated with 100 nM CDDO-Im. Orange: cells in media containing 0.35% DMSO. Yellow: cells in media containing no DMSO. (TIF) [file pone.0044686.s002.tif]

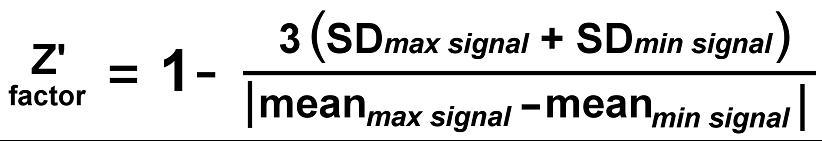

Supplement: Figure S3 — Calculation formulation of Z′ factor. (JPG) [file pone.0044686.s003.jpg]
