# Supplementary material for: Implementation of a High-Throughput Screen for Identifying Small Molecules to Activate the Keap1-Nrf2-ARE Pathway
Source: PLoS One. 2012 Oct 8;7(10):e44686. doi: 10.1371/journal.pone.0044686 (PMC3466241; doi:10.1371/journal.pone.0044686)
Supplement: Table S1 — Oligonucleotide sequences for primers specific for mouse β-actin and Nqo1. (DOCX) [file pone.0044686.s004.docx]

**Table S1.**

**Table S1.** Oligonucleotide sequences for primers specific for mouse β-actin and Nqo1

| Gene | Forward | Reverse |
| --- | --- | --- |
| β-actin | TGACCGAGCGTGGCTACAG | GGGCAACATAGCACAGCTTCT |
| Nqo1 | TATCCTTCCGAGTCATCTCTAGCA | TCTGCAGCTTCCAGCTTCTTG |
